# Retinal Vessel Analysis and Microvascular Abnormalities in Patients with Philadelphia-Negative Chronic Myeloproliferative Neoplasms

**DOI:** 10.3390/jcm13082232

**Published:** 2024-04-12

**Authors:** Anna Maria Roszkowska, Rossana Leanza, Emanuela Aragona, Ludovica Gargiulo, Angela Alibrandi, Alessandro Arrigo, Adele Bottaro, Paola Barone, Fabio Stagno, Alessandro Allegra

**Affiliations:** 1 Ophthalmology Unit, Department of Biomedical Sciences, University of Messina, 98100 Messina, Italy; anna.roszkowska@unime.it (A.M.R.); ludovicagargiulo@gmail.com (L.G.); 2 Ophthalmology Department, Faculty of Medicine and Health Sciences, Andrzej Frycz Modrzewski Kraków University, 30-705 Kraków, Poland; 3 Hematology Unit, Department of Human Pathology in Adulthood and Childhood “Gaetano Barresi”, University of Messina, Via Consolare Valeria, 98125 Messina, Italy; leanzaross@gmail.com (R.L.); adelebottarp15@gmail.com (A.B.); baronepaola2903@gmail.com (P.B.); fabio.stagno@unime.it (F.S.); 4 Istituto di Ricovero e Cura a Carattere Scientifico San Raffaele Scientific Institute, 20132 Milan, Italy; aragona.emanuela@hsr.it (E.A.); arrigo.alessandro@hsr.it (A.A.); 5 Unit of Statistical and Mathematical Sciences, Department of Economics, University of Messina, 98100 Messina, Italy; angela.alibrandi@unime.it

**Keywords:** Philadelphia-negative chronic myeloproliferative neoplasms, thrombosis, polycythemia vera, essential thrombocythemia, primary myelofibrosis, thrombotic risk, prognosis, retinal exams, optical coherence tomography

## Abstract

**Background**: Philadelphia-negative chronic myeloproliferative neoplasms are a group of clonal hematopoietic disorders including polycythemia vera, essential thrombocythemia, and primary myelofi-brosis. These neoplasms are characterized by an increased risk of thrombotic complications. Several studies have highlighted that the study of vessels of the retina offers the opportunity to visualize, in vivo, the damage to microcirculation that is common in various systemic pathologies. **Methods:** in our study, forty patients underwent an ophthalmological examination, using non-invasive imaging tech-niques, for analyses of their retinal vascularization. The objective was to correlate the findings ob-tained from this study of the retina with different markers of thrombotic risk, to demonstrate the usefulness of studying retinal vessels as a possible new prognostic biomarker of thrombotic risk in patients affected by Philadelphia-negative chronic myeloproliferative neoplasms. **Results:** retinal imaging demonstrated changes in the microcirculation, with a reduced vascular density of the deep and superficial capillary plexuses with respect to a normal group, and a correlation between retinal changes and blood parameters. **Conclusions:** additional research will allow us to determine whether retinal changes in individuals with chronic myeloproliferative neoplasms could be predictive of the development of thrombotic events in these subjects.

## 1. Introduction

Philadelphia-negative (Ph−) chronic myeloproliferative neoplasms are clonal diseases that arise from a pluripotent hematopoietic stem cell. They include, among others, polycythemia vera (PV), essential thrombocythemia (ET), and primary myelofibrosis (PMF) [1].

In addition to their possible evolution into acute leukemia, myeloproliferative neoplasms are characterized on a clinical level by vascular risk, in terms of thrombotic and/or hemorrhagic complications [2,3].

Driver mutations have been identified in chronic myeloproliferative diseases. In some cases, the V617F mutation of the JAK2 gene is present in the neoplastic cell. Other driver mutations are CLR (calreticulin) and MPL (thrombopoietin receptor). Patients who do not have mutations in any of the three genes mentioned are defined as triple-negative [4]. Finally, further mutations have recently been identified that concern important gene types, including epigenetic modifiers [5,6]. 

These mutations have been variously associated with thrombotic risk. In fact, for example, the risk of thrombosis, for the same number of platelets, is greater in patients with JAK2 mutations than those with CALR mutations. Therefore, in addition to the number of platelets, the type of mutation is also able to influence the thrombotic risk. The quality of the mutation, i.e., the so-called allelic pattern, is also important: in particular, a relevant JAK2 mutant burden is accompanied by an increase in thrombotic risk [7,8].

The evaluation of vascular alterations is extremely important in this type of patients. Since the eye offers direct visualization of the retinal microcirculation, it is possible to non-invasively evaluate the retinal vasculature through high-quality imaging and the assistance of reliable and validated software, and, consequently, visualize damage to the microcirculation in vivo. The vascularization of the retina can be easily observed using fundus photography, video recording, and tomographic technology, which is becoming a useful screening tool for cerebrovascular and cardiovascular diseases, given that several studies have demonstrated a correlation between the calibers of the retinal vessels and cardiovascular risk [9,10]. 

The study of retinal vessels has been used in the past to evaluate cardiovascular status in various sets of patients, although not in patients with chronic myeloproliferative diseases. A study conducted in diabetic patients demonstrated that narrower retinal arteriolar diameters and wider retinal venular diameters were associated with higher mortality [11,12,13].

In a different experiment, the arteriole/venule ratio (AVR) was calculated as an index of arteriolar narrowing, wherein a smaller AVR indicates greater narrowing. The relative risk of stroke increased as the arteriole/venular ratio decreased. The associations were similar for ischemic strokes and strokes in subjects with hypertension, with or without diabetes [14]. A further area of research concerned the relationship between atherosclerosis of the retinal arteries with the extension and severity of coronary heart disease, while another study confirmed the correlation between the diameter of retinal vessels and known cardiovascular risk factors [15,16]. It has even been shown that changes in the diameter of retinal vessels predict the risk of cardiovascular disease and stroke mortality, independent of traditional factors [16].

Finally, patients with age-related macular degeneration and drusen are more likely to have myeloproliferative neoplasms. Researchers have found that myeloproliferative neoplasms with drusen had higher levels of chronic low-grade inflammation than myeloproliferative neoplasms without retinal degeneration. This finding may suggest that systemic chronic low-grade inflammation contributes to or possibly initiates the creation of drusen. Age-related macular degeneration is more likely to occur when myeloproliferative neoplasms cause chronic low-grade inflammation that leads to the production of drusen and further episodes of low-grade inflammation [17].

The purpose of our study was to evaluate the existence of retinal alterations between a population of patients with Philadelphia-negative chronic myeloproliferative neoplasms and a historical healthy control population. Furthermore, we attempted to correlate the findings obtained from the retinal study with thrombotic risk markers. The aim was to highlight which data could provide an adequate prognostic assessment of vascular risk in order to possibly validate the retinal study as a useful marker of thrombotic risk in patients with Philadelphia-negative chronic myeloproliferative neoplasia.

## 2. Materials and Methods

The study was conducted on patients suffering from Ph− chronic myeloproliferative neoplasms belonging to the Hematology Unit of the Policlinico di Messina (Italy), both upon their first diagnosis and at follow-up assessments. The study was conducted according to the guidelines of the Declaration of Helsinki, and approved by the Ethics Committee of Messina (resolution n. 200 of 31 January 2024).

In total, 40 patients were analyzed: 19 were female and 21 were male, with an average age of 67 years, and all were affected by Ph− chronic myeloproliferative neoplasms.

Specifically, 18 patients were diagnosed with essential thrombocythemia, treated with hydroxyurea, acetylsalicylic acid (ASA), hydroxyurea + ASA, or peginterferon + ASA; 15 were diagnosed with polycythemia vera, which was being treated with hydroxyurea + ASA, ASA, or Ruloxitinib; and the remaining 7 had primary myelofibrosis, which was being treated with hydroxyurea + ASA or Ruloxitinib. The patients’ characteristics are summarized in Table 1.

### 2.1. Inclusion Criteria

- Had an established diagnosis of chronic myeloproliferative neoplasia;
- Aged over 18 years;
- Provided informed consent.

### 2.2. Exclusion Criteria

- Affected by congenital hypercholesterolemia;
- Affected by a known serious cardiovascular pathology;
- Affected by a known thrombophilic state;
- Under 18 years old.

The patients underwent an initial visit for the collection of medical history data. On the same occasion, after signing an informed consent form, they were subjected to blood sampling for the measurement of markers of glucose and lipid metabolism and all of the indices aimed at evaluating the state of the myeloproliferative disease. Indices suitable for evaluating the thrombotic risk such as the Framingham score and the Precard score were taken into consideration, the blood parameters were recorded, and the genetic analysis was carried out for the driver mutations of chronic myeloproliferative diseases.

Furthermore, the patients underwent a full ophthalmological examination in the Ophthalmology Unit with optical coherence tomography (OCT) for the assessment of their retinal vessels. 

The ophthalmologic examination included uncorrected and best corrected visual acuity (UCVA and BCVA) assessments using a standard ETDRS chart, refraction analysis using an autorefractometer (Topcon Corporation, Tokyo, Japan), slit-lamp biomicroscopy analyses of the anterior and posterior segments of the eye, Goldmann applanation tonometry analysis, and ophthalmoscopy analysis. OCT was performed using swept-source OCT (DRI OCT Triton, Topcon Corporation, Tokyo, Japan). 

The exclusion criteria for the OCT evaluation were any other type of retinal or optic nerve diseases, ophthalmologic surgery within the last three months, and any uncontrolled systemic condition that could potentially affect the analyses. 

OCT images were obtained using 4.5 × 4.5 mm^2^ acquisitions. Automatic segmentations of the superficial capillary plexa (SCP), deep capillary plexa (DCP), and choriocapillaris (CC) were obtained from the OCT acquisitions. Each segmentation was carefully inspected and, if necessary, manually corrected by two expert ophthalmologists (A.A., E.A.), taking only high-quality images (Topcon quality index > 80) into consideration. All reconstructions were loaded in ImageJ software (Version 1.15.0) (https://imagej.net/Welcome accessed on 5 February 2024) to calculate the vessel density (VD). First of all, the OCT reconstructions were binarized using the following pipeline: image loading -> adjust threshold -> automatic mean threshold. An in-house script was used to calculate the VD, interpreted as the ratio of white pixels to black. The foveal avascular zone was manually segmented and excluded. 

A historical group of 30 healthy subjects comparable in age and sex was used as a control group. All of the subjects were screened for any systemic or ocular disorders before their inclusion, particularly for the presence of systemic or retinal vasculopathies. Moreover, an exclusion criterion was any ocular surgery performed at least six months before the inclusion date. 

The biological material was stored at −80 °C until assayed.

### 2.3. Statistical Analyses

The data obtained were subjected to verification and quality control tests, and subsequently to descriptive and inferential statistical analyses.

A data comparison was carried out between the study population and the historical control population. Furthermore, a comparison was carried out between patients with myeloproliferative neoplasia affected and not affected by thrombotic events.

**Figure 1:**
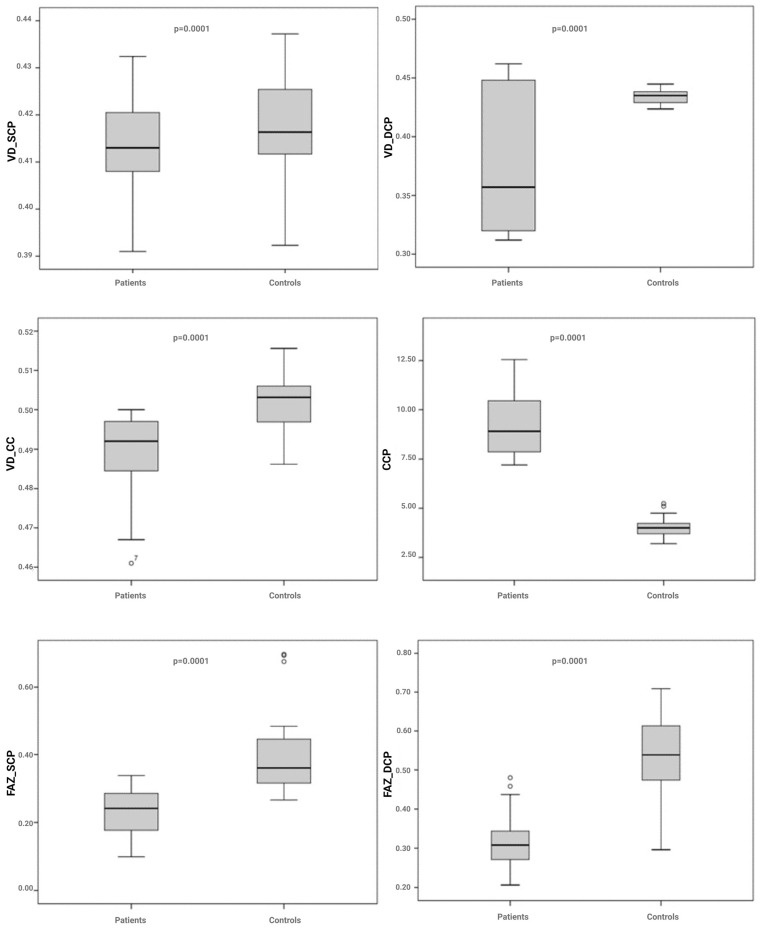
Boxplot of OCT vascular parameters evaluated in the two study populations. VD_SCP: vessel density of the superficial capillary plexus; VD_DCP: vessel density of the deep capillary plexus; VD_CC: vessel density of the choriocapillaris; VT-SCP: vessel tortuosity of the superficial capillary plexus; VT-DCP: vessel tortuosity of the deep capillary plexus; CCP: choriocapillaris porosity; FAZ_SCP: foveal avascular zone of the superficial capillary plexus; FAZ_DCP: foveal avascular zone density of the deep capillary plexus.

Continuous data were expressed as the mean ± S.D.: differences between groups were evaluated using parametric tests (Analysis of Variance (ANOVA) with post hoc multiple comparisons (Bonferroni)) or non-parametric tests (Kruskal–Wallis test) for multiple independent samples. Bonferroni’s correction was applied in the multiple comparison test, so that only a *p*-value lower than the 0.050 level, divided by the possible number of comparisons that could be performed (alpha-adjusted), was considered statistically significant. In the comparison between groups, we considered essentially 4 aspects that generate multiplicity effects: density (vessel density—VD, and choriocapillaris vascular density—VD CC), choriocapillaris porosity (CCP), vessel tortuosity (VT), and foveal avascular zone (FAZ). Bonferroni’s correction is necessary to control the effect of multiplicity. More specifically, the Bonferroni-adjusted alpha level is 0.0125 per test (0.050/4); so, in Figure 1, we only considered *p*-values < 0.0125 to be statistically significant (all comparisons except VD_SCP).

Correlation (Pearson correlation) and regression (Linear regression) analyses were performed to evaluate possible associations between the clinical variables studied. This was a secondary aim of our research article.

A separate analysis was carried out to evaluate the impact of the concentrations of the studied markers on the response to treatment.

Results were considered significant for *p*-values < 0.05. Statistical analysis was performed using the Statistical Package for the Social Sciences (SPSS) software 18.

The imaging evaluation was carried out at the S. Raffaele Institute in Milan using techniques that allowed the detection of possible correlations between the imaging and biological information.

### 2.4. OCT Parameters

In this study, the differences in OCT parameters related to retinal and choroidal vessels between healthy subjects and those affected by Ph− chronic myeloproliferative neoplasms were examined. The considered parameters comprised vessel density (VD), choriocapillaris vascular density (VD CC), choriocapillaris porosity (CCP), vessel tortuosity (VT), and the foveal avascular zone (FAZ). Vessel density (VD) is a measurement that expresses the percentage ratio between the vascular surface and the nonvascular retinal surface [18]. The choriocapillaris vascular density (VD CC) measures the thin but dense vascular monolayer located along the internal choroid, adjacent to Bruch’s membrane [19]. Several histopathological studies have demonstrated the existence of a correlation between anomalies in the integrity of the CC and ocular pathologies such as AMD (age-related macular degeneration), diabetic retinopathy, and uveitis [20].

The choriocapillaris porosity (CCP) is the percentage calculation of flow voids compared to the entire choriocapillaris surface. Flow voids are voids detected in the choriocapillaris surface via OCT imaging [21,22,23,24]. 

Vessel tortuosity (VT) describes the ratio between the length of the vessel being taken into consideration and the maximum distance used to reach the two extremes. Given a parametric curve with endpoints A and B, its tortuosity is greater the higher the ratio between the length of the curve and the distance between the two points [25].

The foveal avascular zone (FAZ) is a vascular finding that is particularly useful in healthy subjects. This is an area without vascularization, with a diameter between 500 and 600 µm, located in the foveal region and surrounded at its edges by the interconnections of capillary endings. This area reflects the health status of the retinal capillary circulation and its measurement provides valid assistance in the diagnosis and management of retinal vascular pathologies. Several studies have correlated the FAZ with the central foveal thickness, revealing an inverse proportionality relationship and therefore suggesting that a thicker retina is characterized by a smaller FAZ, while a thinner retina has a wider FAZ. This is justified by the fact that a thicker retina sends a greater metabolic demand and, consequently, a greater vascular supply with a reduction in the size of the FAZ [26].

It has been demonstrated that OCT can efficiently provide both a quantitative and qualitative assessment of the normal FAZ, an assessment that in the past has proven difficult to obtain with standard methods such as fluorescein angiography.

In vascular occlusions, for example, thanks to OCT, it is possible to carry out a careful analysis of the FAZ and its changes, detecting not only frequent increases in its size but also singular findings such as macular–foveal capillaries (MFCs). The latter comprise an area of complete or partial absence of the FAZ, highlighted in OCT as a polygonal or rectilinear capillary network that, crossing the macular region centrally or eccentrically, connects to the surrounding retinal capillary bed [27].

## 3. Results

The results of the statistical analyses demonstrate that in patients affected by myeloproliferative neoplasms, the variables examined differ significantly on average, except the VD_SCP. 

As for the CCP, i.e., the percentage of flow voids compared to the entire choriocapillaris surface detected via OCT imaging, the results show that in patients suffering from Ph− chronic myeloproliferative neoplasms, the flow voids in the choriocapillaris are significantly higher (9.2106) compared to healthy reference subjects (4.0390) (Figure 1).

Furthermore, our data show that in patients suffering from chronic Ph− myeloproliferative diseases, the vessel tortuosity values, both in the superficial and deep capillary plexuses, are significantly higher than in healthy reference subjects.

**Figure 2:**
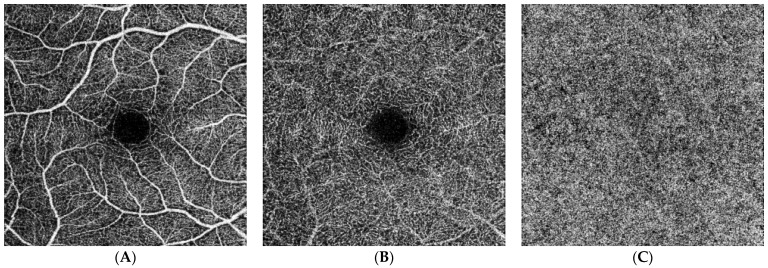
OCT reconstructions of a healthy control subject. These en face OCT images represent the normal perfusion signal and vasculature profiles both for the SCP (**A**) and DCP (**B**). In addition, the en face OCT imaging reconstructs well the porous structure of the CC (**C**).

**Figure 3:**
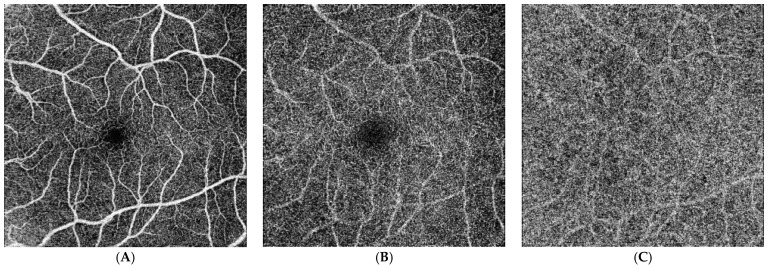
OCT findings in patients affected by essential thrombocythemia. En face OCT images show the perfusion characteristics and vasculature profile to be within normal limits for the SCP (**A**). The DCP (**B**) is characterized by perfusion reduction and wide alterations of the capillary network. In addition, the en face OCT images show reduced perfusion affecting the CC (**C**). Reduced FAZs both in the SCP and DCP are evident when compared with the healthy controls.

Additionally, the data show that in patients suffering from Ph− chronic myeloproliferative neoplasms, the FAZ is smaller, both in the superficial and deep capillary plexuses, as compared to healthy reference subjects (Figure 2 and Figure 3).

The correlations between the ocular parameters and hematological variables calculated with Pearson correlation and Linear regression evidenced statistically significant correlations between the erythrocytes and IOP RE and between the erythrocytes and IOP LE (Table 2).

Statistically significant correlations were found between the Precard score and the Framingham score, as well as between the Precard score and creatinine. Worthy of noting is the correlation between the Framingham score and creatinine, with a statistical significance value of 0.001.

Other significant correlations were found between Precard score and hemoglobin and between the Precard score and hematocrit. Other notable correlations were found between the Framingham score and FDPs, as well as between the Framingham score, hemoglobin, and hematocrit. Furthermore, a correlation was evidenced between the Framingham score and the platelets.

Finally, no statistical significance emerged for correlations between the JAK2 and ophthalmological parameters (Table 3).

## 4. Discussion

It is well known that solid tumors are associated with thromboembolic complications [28], but recent studies have shown that the incidence of thrombosis may be equally high, if not even higher, in patients with malignant hematological disorders [29,30,31,32].

The pathogenesis of the circulatory complications common in Ph− chronic myeloproliferative neoplasms [3] is not yet fully known, but the mechanical effects of the high cell count probably contribute to increasing blood viscosity, in addition to the effects being mediated by the secretion of prothrombotic mediators from neoplastic cells, by the expression of neoplastic adhesion molecules, and by the adhesion of cells to a hyper-stimulated vascular endothelium [33,34,35].

An important role in the pathogenesis of thrombosis in myeloproliferative diseases could be played by adhesion molecules. It has been shown that adherence increases in such patients; in particular, in polycythemia vera, there is an overexpression of Lu/BCAM, two glycoprotein isoforms of the immunoglobulin superfamily that represent the receptors for the α5 chain of laminin [36].

A further cause of retinal alterations in patients with chronic myeloproliferative disease could be complement dysregulation, capable of playing a fundamental role in the formation of drusen [37]. Drusen are extracellular deposits of lipids, proteins, and cellular debris that are found within the layers of the retina and appear as small yellow deposits upon ophthalmological examination. Specifically, they reside between the basal lamina of the retinal pigment epithelium and the internal layer of Bruch’s membrane and are observed physiologically with advancing age. However, their accumulation in the eye, especially in the macula, is a significant risk factor for the development of age-related macular degeneration. Drusen accumulation is associated with a higher neutrophil/lymphocyte ratio, indicating a higher level of chronic low-grade inflammation, and chronic myeloproliferative neoplasms can be included in the broader category of inflammatory pathologies: in fact, a model has been developed that describes chronic inflammation as both a source of origin and an evolution of the development of drusen [38,39,40].

Finally, the increase in oxidative stress typical of chronic myeloproliferative diseases could also be important in this sense [41,42].

Beyond the possible pathogenetic moments, thrombosis, either arterial or venous (deep-vein thrombosis, pulmonary embolism, and thrombosis in uncommon sites), is one of the main clinical factors given its substantial effect on morbidity and mortality [43]. 

New combined clinical and cytogenetic scores such as the Mutation-Enhanced International Prognostic Score System for Transplantation-Age Patients with Primary Myelofibrosis, genetically derived prognostic scoring systems such as the GIPSS, and the Myelofibrosis Secondary to PV and ET- Prognostic Model have been formulated that manage the identification of high-risk patients [44]. However, numerous clinical needs persist unmet, notwithstanding the enormous quantity of existing genetic and molecular data. 

In our study, we highlighted a significant difference in the microvascular status of the retina between patients suffering from Ph− chronic myeloproliferative neoplasms as compared to healthy subjects. 

Previous experimental studies have highlighted the retina’s vessels as biomarkers of various systemic pathologies linked to alterations of the microcirculation, including diabetes, hypertension, Alzheimer’s disease, and various hematological diseases [45,46].

In our patients, retinal imaging demonstrated changes in the microcirculation, as evidenced by the reduced vascular density of the deep and superficial capillary plexuses. This might suggest that there is reduced perfusion in these capillary regions.

Furthermore, our patients with Ph− chronic myeloproliferative neoplasms exhibited other similar vascular changes, as seen by decreases in their FAZs. Another modified parameter was the porosity values of the choriocapillaris, probably due to the decreased flow signals in the capillary regions that were investigated. The rise in the vascular tortuosity parameter may be traceable to the initial changes that the vascular network experiences in different retinopathies, such as those caused by diabetes and hypertension [47].

Moreover, in our study, it was feasible to verify the existence of correlations between the clinical indicators, which represent each patient’s cardiovascular health, and the hematological measures mentioned above thanks to the statistical analyses carried out.

Specifically, it was found that there is a statistically significant correlation between the Precard cardiovascular risk score and the corrected visual acuity of the right eye, which relates to HDL cholesterol. It has been established that HDL lipoproteins possess anti-thrombotic, anti-inflammatory, antioxidant, and anti-apoptotic characteristics, all of which play varying roles in causing harm to the microcirculation [48]. The patients’ reported fuzzy vision may have been brought on by rheological abnormalities that resulted in microcirculatory problems.

It was also confirmed that there is a strong association between the quantity of leukocytes and erythrocytes and the intraocular pressure of the right (IOP RE) and left (IOP LE) eyes. It can be supposed that patients with MPNs often experience vasoconstriction, and microvascular changes might alter ocular pressure. On this basis, we might hypothesize that the VT and FAZ area changes might be signs of retinal vascular remodeling occurring in these patients. However, we have to acknowledge that all imaging methodologies are prone to possible artifacts [49]. These may be related to the patients’ collaboration and blood flow changes occurring in systemic diseases. For this reason, we cannot exclude possible effects related to arterial blood pressure and blood viscosity changes occurring in patients with myeloproliferative disorders. Anyhow, the statistically significant differences observed between the patients and healthy controls support the hypothesis that OCT can be used as a non-invasive diagnostic approach in this kind of patients.

Using the Framingham and Precard scores, an estimation of the cardiovascular risk of the patients undergoing evaluation was also carried out. The rise in blood viscosity and hemoconcentration brought on by the trilinear cellular hyperplasia that occurs in MPNs is predictive, as seen by their statistically significant link with hemoglobin levels, hematocrit, and platelets, of a ten-year rise in the risk of cardiovascular events. Additionally, a link between the same scores and the serum creatinine levels was seen. Another study that established a correlation between poor renal function and elevated cardiovascular risk also showed that this correlation exists both in patients going through the early stages of nephropathy and in those who are terminally uremic [50].

Conversely, a negative Pearson coefficient value (−0.335) indicated an inverse relationship between the Framingham score and fibrinogen degradation products (FDPs); these products play a crucial role in the fibrinolytic process, counteracting the thrombotic predisposition and lowering the risk of cardiovascular events by interfering with thrombin-mediated platelet activation and fibrin polymerization [51].

There has been additional evidence of an inverse relationship between hemoglobin levels and the CALR gene mutation. In fact, patients with primary myelofibrosis who have the same mutation have a significantly longer overall survival rate compared to subjects positive for other mutations (JAK2, V617F, or MPL). Clinically, patients with essential thrombocythemia who are positive for CALR mutations exhibit a lower incidence of thrombotic events [51,52].

The correlation in question in this study is confirmed, as was expected: endothelial dysfunction, platelet hyperactivity, oxidative stress, and low-grade inflammation are some of the events that affect the vascular wall as a result of the metabolic environment that characterizes type 2 diabetes mellitus and includes insulin resistance, hyperglycemia, and the release of excess free fatty acids, in addition to other metabolic anomalies. The primary factor that triggers the inflammatory reactions linked to circulatory problems, disturbs the delicate balance between vasoconstrictors and vasodilators, and inhibits anticoagulant mechanisms is specifically endothelial dysfunction. Hence, elevated D-dimer levels indicate a cascade of events that cause microvascular damage and a propensity for thromboembolism when blood sugar levels are high [53].

To sum up, our study shows that non-invasive retinal imaging techniques are valuable for analyzing the state of the microcirculation in individuals with Ph− chronic myeloproliferative neoplasms.

Verification of our results may offer a helpful tool for predicting thrombotic events in this patient population, since retinal arteries are easily examined.

Finally, our data may potentially offer new perspectives for future investigations. In fact, we plan to further investigate the relationship between changes in retinal vessels and endothelial dysfunction by evaluating various molecular markers of adhesion. These markers include thrombomodulin, a membrane glycoprotein expressed by the vascular endothelium, p-Selectin, an adhesion molecule found in platelets and endothelial cells, sVCAM, and sICAM [54,55].

Our results strongly support the idea that in individuals with Ph− chronic myeloproliferative neoplasms, microvascular dysfunction plays a more significant role in the pathophysiology of thrombosis.

However, grouping the different nosological entities that make up Ph− chronic myeloproliferative neoplasms is likely a shortcoming of our study. Indeed, the various risk factors—such as elevated hematocrit and hemoglobin—have varying weights in relation to different illnesses. Moreover, the various treatments that were employed undoubtedly changed the values of the variables that were looked at. More participants and a study aimed at assessing retinal changes over time will enable a more thorough analysis and more reliable findings.

Thus, prospective future research on this crucial subject is required, hopefully providing answers to the questions of whether therapeutic approaches affecting myeloproliferative disorders may reverse retinal vascular alteration and whether the assessment of retinal alterations could inform clinical decision making.

Further investigations will be necessary to determine whether treatment with new drugs, such as JAK 2 inhibitors, can restore retinal normalcy and whether retinal evaluation will influence therapeutic decision making.

Furthermore, it will be crucial to perform a prospective assessment and determine whether patients with more severe retinal changes will also experience more serious or precocious thrombotic events. This evidence would allow researchers to investigate the effects of using data related to retinal circulation in prognostic scores.

## Figures and Tables

**Table 1:** Patients in the study: essential thrombocythemia (ET), polycythemia vera (PV), primary myelofibrosis (PM), Ruxolitinib (RUX), hydroxyurea (HY), acetylsalicylic acid (ASA), PEG-INTERFERON (PegIFN). Data are expressed as the median and standard deviation.

Diagnosis	n. Pat.	Age	Driver Mutations	Treatm.	Precard	Framingham Score	Fibrinogen mg/dL	FdPs mcg/dL	D-Dimer ng/L	Hb g/dL	Hct %	WBC Mmc	PLT Mmc	Comorb.
ET	18	64 ± 11.53	JAK2 12 MPL 4 CARL 1 Triple neg. 1	HY + ASA 12 ASA 5 PegIFN + ASA 1	(range 1–9)	4 ± 10.27 (range 0.7–20.1%)	297.5 ± 54.08 (range 200–436)	3 ±1.70 (range 1–8)	201 ±155.96 (range 70–700)	13.5 ± 1.68 (range 10.8–17.5)	39.8 ± 4.38 (range 31.6–47.9)	7745 ± 3188 (range 4240–17,400)	324.000 ± 1,380,000 (range 503,500 ± 252,959)	Hypert. 7 Atrial Fibr. 1 Diabetes 1 Thromb. 5
PV	15	73 ± 7.6	JAK2 14 Triple neg. 1	HY + ASA 12 Runx 1 ASA 2	(range 2–14)	16.35 ± 5.54) (range 4.7 ± 20.4)	238 ± 70.4 (range 147–400)	2.5 ± 1.17 (range 0–4)	217 ± 269 (range 115–1216)	14.4 ± 1.83 (range 11.5–17.3)	43.3 ±5.04 (range 32.4–49.4)	9000± 4678 (range 4840–23,600)	489,000 ± 209,727 (range 88,000 ± 740,000)	Hypert. 12 Atrial Fibr. 2 Diabetes 1 Thromb. 6
PM	7	67 ± 7.1	JAK2 4 CALR 1 Triple neg. 2	HY + ASA 5 Runx 2	(range 2–14)	14.3± 5.4 (range 5.9–19)	265 ± 64.3 (range 205–410)	3± 0.9 (range 1–4)	379 ± 156.3 (range 170–690)	11.8 ± 1.53 (range 11.1–15.5)	34.6 ± 3.75 (range 31.3–42.7)	6340 ± 2663 (range 4340 ± 13,300)	406,000 ± 317,601 (range 281,000–1,166,000)	Hypert. 5 Thromb. 1

**Table 2:** Correlations highlighted between intraocular pressure and the hematological variables. IOP RE: intraocular pressure in the right eye; IOP LE: intraocular pressure in the left eye.

Variable 1	Variable 2	Pearson Correlation	*p*
Erythrocytes	IOP RE	0.426	0.01
Erythrocytes	IOP LE	0.419	0.01

**Table 3:** Correlations highlighted between the hematological variables and prognostic scores.

Variable 1	Variable 2	Pearson Correlation	*p*
Precard score	Hb	0.371	0.01
Precard score	Ht	0.392	0.01
Precard score	Creatinine	0.324	0.04
Framingham score	FDPs	−0.335	0.03
Framingham score	Hb	0.328	0.03
Framingham score	Ht	0.402	0.01
Framingham score	Platelets	−0.384	0.01
Framingham score	Creatinine	0.498	0.001
Erythrocytes	White blood cells	0.457	0.003
Platelets	Ht	−0.401	0.010

## Data Availability

The data presented in this study are available on request from the corresponding author.
